# Factors associated with timely treatment of malaria in the Brazilian Amazon: a 10-year population-based study

**DOI:** 10.26633/RPSP.2017.100

**Published:** 2017-07-20

**Authors:** Isac da S. F. Lima, Elisabeth C. Duarte

**Affiliations:** 1 Postgraduate Tropical Medicine Program, Tropical Medicine Department School of Medicine, University of Brasília Brasília Brazil Postgraduate Tropical Medicine Program, Tropical Medicine Department, School of Medicine, University of Brasília, Brazil.; 2 Tropical Medicine Department School of Medicine, University of Brasília Brasília Brazil Tropical Medicine Department, School of Medicine, University of Brasília, Brasília, Brazil.

**Keywords:** Malaria, time-to-treatment, Brazil, Malaria, tiempo de tratamiento, Brasil, Malária, tempo para o tratamento, Brasil

## Abstract

**Objective.:**

To identify factors associated with timely treatment of malaria in the Brazilian Amazon. Malaria, despite being treatable, has proven difficult to control and continues to be an important public health problem globally. Brazil accounted for almost half of the 427 000 new malaria cases notified in the Americas in 2013.

**Methods.:**

This was a cross-sectional study using secondary data on all notified malaria cases for the period from 2004 – 2013. Timely treatment was considered to be all treatment started within 24 hours of symptoms onset. Multivariate logistic regression was used to identify independent factors associated with timely treatment.

**Results.:**

The proportion of cases starting treatment on a timely basis was 41.1%, tending to increase in more recent years (OR = 1.40; 95%CI: 1.37 – 1.42 in 2013). Furthermore, people starting within < 24 hours were more likely to: reside in the states of Rondônia (OR = 1.50; 95%CI: 1.49 – 1.51) or Acre (OR = 1.53; 95%CI: 1.55 – 1.57); be 0 – 5 years of age (OR = 1.39; 95%CI: 1.34 – 1.44) or 6 – 14 years of age (OR = 1.34; 95%CI: 1.32 – 1.36); be indigenous (OR = 1.41; 95%CI: 1.37 – 1.45); have a low level of schooling (OR = 1.20; 95%CI: 1.19 – 1.22); and be diagnosed by active detection (OR = 1.39; 95%CI: 1.38 – 1.39).

**Conclusion.:**

In the Brazilian Amazon area, individuals were more likely to have timely treatment of malaria if they were young, residing in Acre or Rondônia states, have little schooling, and be identified through active detection. Identifying groups vulnerable to late treatment is important for preventing severe cases and malaria deaths.

## RESUMEN

**Objetivo.:**

Determinar los factores asociados con el tratamiento oportuno de la malaria en la Amazonia brasileña. La malaria, a pesar de que es tratable, ha resultado difícil de controlar y sigue siendo un problema importante de salud pública mundial. En Brasil se notificaron casi la mitad de los 427 000 nuevos casos de malaria en la Región de las Américas en el 2013.

**Métodos.:**

Se realizó un estudio transversal que utilizó datos secundarios de todos los casos notificados de malaria en el período 2004–2013. Se entendió como tratamiento oportuno todo tratamiento iniciado en las 24 horas posteriores a la aparición de los síntomas. Para determinar los factores independientes asociados con el tratamiento oportuno, se usó el método de regresión logística multifactorial.

**Resultados.:**

La proporción de casos en los que se inició el tratamiento oportunamente fue de 41,1%, con una tendencia ascendente en los últimos años (razón de posibilidades [OR] = 1,40; IC 95%: 1,37 – 1,42 en el 2013). Además, en las personas que comenzaron el tratamiento menos de 24 horas después de la aparición de los síntomas era mayor la probabilidad de que residieran en los estados de Rondônia (OR = 1,50; IC 95%: 1,49 – 1,51) o Acre (OR = 1,53; IC 95%: 1,55 – 1,57); también era mayor la probabilidad de que tuvieran entre 0 y 5 años (OR = 1,39; IC 95%: 1,34 – 1,44) o entre 6 y 14 años (OR = 1,34; IC 95%: 1,32 – 1,36); fueran indígenas (OR = 1,41; IC 95%: 1,37 – 1,45); tuvieran un nivel bajo de escolarización (OR = 1,20; IC 95%: 1,19 – 1,22) y hubieran sido diagnosticadas por detección activa (OR = 1,39; IC 95%: 1,38 – 1,39).

**Conclusiones.:**

En la zona de la Amazonia brasileña, era más probable que las personas que iniciaban oportunamente el tratamiento contra la malaria fueran jóvenes, residieran en los estados de Acre o Rondônia, tuvieran un nivel bajo de escolarización y fueran detectadas mediante la detección activa. La identificación de los grupos vulnerables al tratamiento tardío es importante para prevenir los casos graves y las muertes por malaria.

## RESUMO

**Objetivo.:**

Identificar os fatores associados ao tratamento precoce da malária na Amazônia brasileira. Embora seja tratável, a malária tem sido difícil de controlar e continua a representar um importante problema de saúde pública em escala mundial. Em 2013, o Brasil registrou quase a metade dos 427.000 novos casos de malária notificados nas Américas.

**Métodos.:**

Este foi um estudo transversal que utilizou dados secundários sobre todos os casos de malária notificados no período de 2004 a 2013. O tratamento precoce foi definido como todo tratamento iniciado nas primeiras 24 horas desde o surgimento dos sintomas. Utilizamos a regressão logística multivariada para identificar fatores independentes associados ao tratamento precoce.

**Resultados.:**

A proporção de casos que iniciaram tratamento precoce foi de 41,1%, tendendo a aumentar em anos mais recentes (odds ratio [OR] = 1,40; IC 95%: 1,37 – 1,42 em 2013). Além disso, as pessoas que iniciaram o tratamento em menos de 24 horas tiveram maior probabilidade de: residir nos estados de Rondônia (OR = 1,50; IC 95%: 1,49 – 1,51) ou Acre (OR = 1,53; IC 95%: 1,55 – 1,57); ter entre 0 e 5 anos de idade (OR = 1,39; IC 95%: 1,34 – 1,44) ou entre 6 e 14 anos de idade (OR = 1,34; IC 95%: 1,32 – 1,36); ser indígena (OR = 1,41; IC 95%: 1,37 – 1,45); ter um baixo nível de escolaridade (OR = 1,20; IC 95%: 1,19 – 1,22); e ser diagnosticado por meio da detecção ativa (OR = 1,39; IC 95%: 1,38 – 1,39).

**Conclusão.:**

Na região da Amazônia brasileira, as pessoas têm uma maior probabilidade de receber tratamento precoce para a malária se forem jovens, residirem nos estados do Acre ou de Rondônia, tiverem um baixo nível de escolaridade e forem identificadas através da detecção ativa. A identificação de grupos vulneráveis ao tratamento tardio é importante para prevenir os casos graves e as mortes decorrentes da malária.

Malaria is a treatable, mosquito-borne (genus *Anopheles*) disease; the lifecycle of its etiologic agents (*Plasmodium sp.*) includes humans and invertebrate hosts. The disease has proven difficult to control and persists as an important public health problem. The World Health Organization (WHO) estimates that 3.3 billion people are at risk of contracting the disease worldwide each year (1). In 2013, WHO reported approximately 198 million new cases of malaria and 584 000 related deaths globally. Of these, approximately 427 000 cases (0.2%) were in the Americas, 178 000 (0.09%) of which were in Brazil (1).

Over the last eight decades, malaria transmission in Brazil has shown marked cyclical variations and various large epidemic periods. In the early 1940s, more than 6 million cases were reported, accounting for 20% of the entire population of Brazil at that time (2). In the 1990s, there was another sharp increase—more than 637 000 cases by 1999—associated with migration to the Brazilian Amazon region (BAR).^3^ Since then, malaria transmission has been more concentrated in this area, accounting for 99.9% of the cases in Brazil (3). A total of 266 348 new malaria cases and 69 malaria deaths were reported in Brazil in 2011, representing reductions of approximately 20% and 9% compared to 2010, respectively (4). Moreover, there was a marked increase in the extension of the malariafree territory: from 15.6% of municipalities with no notified new cases in 2003 – 2004, to 31.7% malaria-free municipalities in 2008 – 2009 (5).

In view of the cyclical history of the disease, sustaining reduced malaria incidence and mortality rates continues to be a challenge. Timely diagnosis and adequate treatment of malaria are of particular relevance in settings like the BAR, which are not very amenable to vector control measures (6). Timely diagnosis and treatment do not only help to prevent hospitalizations and deaths, but also help to control disease transmission by preventing or reducing the appearance of the sexual stages of the parasite (gametocytes) in human hosts, the infective forms to the mosquito vectors (4, 6). Clearly, the effectiveness of malaria treatment depends on the parasite species involved in the infection and the time delay between symptoms onset and the appearance of the sexual stages of the parasite (6).

The Brazilian National Malaria Control Program (PNCM) has stipulated that an important indicator of malaria control is the percentage of cases starting treatment within 48 hours after symptoms onset (7). Nevertheless, based on the parasite’s lifecycle, it is expected that the sooner treatment is begun, the more effective it will be, both for patients and for controlling the disease in the community (8, 9). The aim of this study was to identify factors associated with the timely treatment of malaria in the BAR states where the disease is most prevalent.

## MATERIALS AND METHODS

This was a population-based, crosssectional study using secondary data from all cases of malaria notified in selected states of the BAR in 2004 – 2013.

### Study population

Patients with symptomatic malaria, living in any of the six states of interest to this study (Acre, Amapá, Amazonas, Pará, Rondônia, and Roraima) comprised the study population. The states of Maranhão, Mato Grosso, and Tocantins, although part of the BAR, were not included in the study because they account for only 2.0% of all incident malaria cases reported in the country (10). Although each selected state has distinct economic activities, they share many similarities, such as low population density and a relatively high percentage of rural inhabitants (11).

### Episode of malaria

This study considered all symptomatic and positive malaria tests reported in the states of interest. Additionally, follow-up visits with cure verification slides were excluded, since these were clearly not new. Therefore, the term “malaria incident case” or “episode of symptomatic malaria” was used in this study to mean a “positive malaria test from a symptomatic person.”

### Data source

Data were obtained from the Malaria Epidemiological Surveillance Information System (SIVEP-Malaria), a database managed by the PNCM that collects all malaria tests performed in public or private health services throughout the BAR. In Brazil, notification of malaria is mandatory; therefore, all events must be reported to this information system or to the Notifiable Diseases Information System (SINAN) when the case is present in other areas of Brazil. The data was analyzed according to the patient’s place of residence.

### Study variables

#### Dependent variable.

- Timely treatment. Considered to be any anti-malaria treatment started within 24 hours following the onset of symptoms.

#### Independent variables.

Aggregated as demographics, socioeconomics, and malaria-related variables as follows:

- Demographics. (a) age group: “0–5 years of age,” “6–14 years,” “15–29 years,” “30–59 years,” or “60 years or more;” (b) sex: “female,” “male,” or “not informed;” (c) race/color: “white,” “black/brown,” “yellow,” “indigenous,” or “not informed;” (d) state of residence: “Acre,” “Amapá,” “Amazonas,” “Pará,” “Rondônia,” or “Roraima;” and (e) year of case notification (2004–2013).
- Socioeconomics. (a) level of schooling: “no schooling – incomplete 5^th^ grade,” “complete 5^th^ grade – complete 9^th^ grade,” “partial high-school or beyond,” “not applicable,” or “not informed;” (b) type of occupation: “agriculture,” “traveler/tourism,” “livestock farming/crop production/hunting and fishing/bridge building/mining,” “domestic service,” “prospector,” “others,” or “not informed or not applicable.”
- Malaria-related. (a) type of malaria: “*Falciparum*” (*Falciparum*, F+FG, FG, F+M), “*Vivax*“ (*Vivax*, Non-F), “Mixed” (F+V, V+FG), or “Other” (*Malariae*, *Ovale*); (b) parasite density (graded as number of + sign): “+/2” (< 5 parasites/μl), “+” (5 – 9 parasites/μl), “++” (10 – 100/μl), “+++ or more” (> 100 parasites/μl) or “not informed.” According to the plus system, the more plus signs (+), the higher the parasite density; (c) type of detection: “passive detection” or “active detection.” Passive detection occurs when a patient comes to the facility for malaria testing; active detection occurs when health professionals search for people with malaria symptoms.

The reference categories were chosen considering the number of observations in the category (small categories were avoided) and the expected relationship with the outcome (positive effects, Odds Ratio [OR] > 1, were preferable).

The category *not informed* was created for missing data on “level of schooling,” “parasite density,” and “race/color” variables. All children less than 6 years of age (too young for school) were reclassified into *not applicable* for “level of schooling” and “type of occupation” to avoid any potential misclassification. The reclassification due to missing variables or misclassifications accounted for less than 10% of the malaria cases.

### Data analysis

Analysis was performed on a 10-year (2004 – 2013) population database of all malaria cases in the BAR. Frequencies and percentages for each study variable were calculated. Correlation analysis was subsequently performed using Pearson’s correlation to identify high correlation coefficients between independent variables. Multicollinearity between the outcome and the independent variables was also accessed by Variance Inflation Factor (VIF) and Tolerance (12, 13). Variables showing Tolerance ≥ 0.4 were excluded (12).

In the univariate analysis, each variable previously selected was tested against the dependent variable (timely treatment) and crude odds ratios (OR _(crude)_ ); respective 95% confidence intervals (95%CI) and *P* values were estimated. All variables with a *P* < 0.2 were selected for the next stage of the analysis using multivariable logistic regression models (14). Stepwise was used in order to identify the final model. Adjusted odds ratios (AOR) and respective 95%CI were estimated. At this stage, the critical *P* value was set at < 0.05. This study had high statistical power (n), and as such most statistical tests were significant and the clinical/epidemiological significance will be discussed elsewhere. All analyses were performed using SAS version 9.3 (SAS Institute, Cary, North Carolina, United States).

### Ethics

All ethical criteria regarding the Brazilian National Health Council Resolution No. 196/96 were respected, in particular with regard to confidentiality and non-disclosure of information. This study was approved by the Research Ethics Committees from the Faculty of Medicine, University of Brasilia (Brasilia, Brazil).

## RESULTS

A total of 3 365 718 malaria tests were notified in 2004 – 2013. Of these, 420 were excluded because the date of symptoms onset was missing. Therefore, 3 365 298 cases were considered in the analysis, henceforth referred to simply as “malaria cases.”

Except for the variables level of schooling, type of occupation, and race/color, the completeness of the records averaged over 99%. Around 67.2% of malaria cases were among individuals < 30 years of age; with 34.8% among children < 15 years of age. Most cases were males (62.2%), black/brown (10.3%), and residents of the state of Amazonas (36.4%). The highest percentage of notified malaria cases occurred in 2005 (16.0%), and the lowest, in 2013 (4.4%). Among socioeconomic characteristics, malaria cases occurred mainly among those with no formal education or those who had studied up to 9^th^ grade (65.5%); agriculture was the main professional occupation (20.9%). Among malaria-related characteristics, cases were due mainly to *Plasmodium vivax* infections (80.0%), with very low parasite density (“+/2,” 39.7%), and diagnosed by passive detection (76.5%) (Table 1).

**TABLE 1.** Malaria incidence in the states of the Brazilian Amazon area, 2004 – 2013

	Number of cases	Percentage (%)
Malaria incident cases	3 365 298	100.0
Demographic variables		
Age group		
0 – 5 years	439 804	13.1
6 – 14 years	731 537	21.7
15 – 29 years	1 090 736	32.4
30 – 59 years	991 062	29.5
60+ years	112 159	3.3
Sex		
Female	1 270 279	37.8
Male	2 094 569	62.2
Not informed	450	0.0
Race/color		
White	41 130	1.2
Black/Brown	347 331	10.3
Yellow	7 339	0.2
Indigenous	56 570	1.7
Not informed	2 912 928	86.6
State of residence		
Acre	338 708	10.1
Amapá	179 696	5.3
Amazonas	1 224 876	36.4
Pará	898 511	26.7
Rondônia	558 482	16.6
Roraima	165 025	4.9
Year of case notification		
2004	410 596	12.2
2005	537 690	16.0
2006	500 255	14.9
2007	418 767	12.4
2008	287 083	8.5
2009	284 271	8.5
2010	311 446	9.3
2011	246 383	7.3
2012	221 869	6.6
2013	146 938	4.4
Socioeconomic variables		
Level of schooling		
No schooling – incomplete 5^th^ grade	1 293 003	38.4
Complete 5^th^ grade – complete 9^th^ grade	1 012 232	30.1
Partial high-school or beyond	147 446	4.4
Not applicable	556 583	16.5
Not informed	356 034	10.6
Type of occupation		
Agriculture	703 674	20.9
Tourism	49 868	1.5
Livestock farming/crop production/hunting and fishing/bridge building/mining	146 316	4.4
Domestic services	285 005	8.5
Prospector	143 345	4.3
Other	959 000	28.5
Not informed/not applicable	1 078 090	32.0
Malaria-related variables		
Type of malaria		
*Falciparum*	629 363	18.7
*Vivax*	2 692 900	80.0
Mixed	41 749	1.2
Other	1 286	0.0
Parasite density (grade as number of “+” signs)		
+/2	1 337 308	39.7
+	722 650	21.5
++	1 202 109	35.7
+++ or more	95 474	2.8
Not informed	7 757	0.2
Type of detection		
Passive detection	2 574 840	76.5
Active detection	790 458	23.5

*Source:* Prepared by the authors from study data.

Table 2 shows malaria cases distributed according to the time-to-treatment, classified into three categories: < 24 hours (timely treatment); 24 – 48 hours; and > 48 hours. Approximately 41.1% of malaria cases began treatment within 24 hours, 18.9% within 24 – 48 hours, and 40.0% after 48 hours. In percentage terms, children 5 years of age or younger and 6 – 14 years of age received timely treatment more frequently (46.2% and 45.9%, respectively), than older people (39.5% in the 15 – 29 year age group; around 37% for those 30+ years of age). Starting late treatment was more common among those 30 – 59 years of age (43.9%) and those 60+ years of age (44.1%), demonstrating a clear trend of timely treatment among the younger age groups. This trend, however, was not observed comparing the crude (unadjusted) malaria case distribution through the different categories of sex and year of notification.

**TABLE 2.** Malaria incident cases by time between onset of symptoms and treatment initiation in the states of the Brazilian Amazon area, 2004 – 2013

	Total	Time taken to start treatment (%)^a^		
		< 24 hours (timely)	24 – 48 hours	> 48 hours
Malaria incident cases	3 365 298	41.1	18.9	40.0
Age group				
0 – 5 years	439 804	46.2	19.4	34.4
6 – 14 years	731 537	45.9	19.1	35.0
15 – 29 years	1 090 736	39.5	18.9	41.6
30 – 59 years	991 062	37.4	18.6	43.9
60 years or over	112 159	37.0	18.9	44.1
Sex				
Female	1 270 279	41.8	19.0	39.2
Male	2 094 569	40.6	18.9	40.5
Not informed	450	43.1	20.9	36.0
Year of case notification				
2004	410 596	39.2	17.2	43.6
2005	537 690	41.4	18.0	40.7
2006	500 255	43.4	18.2	38.3
2007	418 767	41.0	19.6	39.5
2008	287 083	40.3	20.4	39.3
2009	284 271	41.7	19.7	38.6
2010	311 446	41.7	19.3	39.0
2011	246 383	39.4	19.9	40.8
2012	221 869	40.9	19.4	39.7
2013	146 938	40.0	20.0	40.0

a Time between first symptoms onset and starting treatment.

Note: Row percentages within each category in the table.

***Source:*** Prepared by the authors from study data.

In the multivariable analysis (Table 3), it was found that malaria cases with timely treatment (versus delayed treatment) were more likely to be in the age groups 6 years of age or less (Odds Ratio [OR] = 1.39; 95% Confidence Interval [95%CI]: 1.34 – 1.44); 6 – 14 years of age (OR = 1.34; 95%CI: 1.32 – 1.36); and 15 – 29 years of age (OR = 1.11; 95%CI: 1.11 – 1.12) than in the group 30 – 59 years. Significant likelihood of timely treatment was also found in the following situations: patient records with self-identification of indigenous race/color (OR = 1.41; 95%CI: 1.37 – 1.45) compared to white; residents of Rondônia (OR = 1.50; 95%CI: 1.49 – 1.51), Acre (OR = 1.53; 95%CI: 1.55 – 1.57), or Roraima (OR = 1.26; 95%CI: 1.25 – 1.27) compared to Pará (though residents of Amazonas and Amapá were less likely); and those notified in the years 2012 (OR = 1.44; 95%CI: 1.42 – 1.47) and 2013 (OR = 1.40; 95%CI: 1.37 – 1.42) compared to those notified in 2004.

Level of schooling was the only socioeconomic variable associated with timely treatment, particularly among people with no schooling or who had completed up to the 5^th^ grade (OR = 1.20; 95%CI: 1.19 – 1.22) compared to those with partial high school education or beyond. Similarly, with regard to malaria-related variables, cases receiving timely treatment, compared to those that did not, were more likely to have been tested and diagnosed through active detection (OR = 1.39; 95% CI: 1.38 – 1.39), compared to passive detection. A sensitivity analysis using exclusively data from 2013 was carried out and all factors associated with timely treatment remained statistically significant. Therefore, these results are evidence that, despite the effect of time in the model, the factors related to timely treatment remain the same.

**TABLE 3.** Factors associated with timely treatment of malaria in the Brazilian Amazon, 2004 – 2013

Categories	Unadjusted			Adjusted^a^		
	Odds ratio (OR)	95% Confidence Interval (CI)	*P* value	Adjusted OR	95% Confidence Interval (CI)	*P* value
Demographic variables						
Age group						
0 – 5 years	1.44	1.43–1.45	< 0.01	1.38	1.36–1.40	< 0.01
6 – 14 years	1.42	1.41–1.43	< 0.01	1.33	1.32–1.34	< 0.01
15 – 29 years	1.09	1.09–1.10	< 0.01	1.11	1.11–1.12	< 0.01
30 – 59 years	1.00	—	—	1.00	—	—
60+ years	0.98	0.97–0.99	< 0.01	0.93	0.92–0.95	< 0.01
Race/color						
White	1.00	—	—	1.00	—	—
Black/Brown	1.13	1.10–1.15	< 0.01	1.15	1.13–1.18	< 0.01
Yellow	1.09	1.03–1.15	< 0.01	1.12	1.06–1.18	< 0.01
Indigenous	1.40	1.36–1.43	< 0.01	1.41	1.37–1.45	< 0.01
Not informed	1.31	1.28–1.34	< 0.01	1.48	1.45–1.52	< 0.01
State of residence						
Acre	1.96	1.94–1.97	< 0.01	1.56	1.55–1.57	< 0.01
Amapá	0.78	0.77–0.79	< 0.01	0.86	0.85–0.87	< 0.01
Amazonas	0.88	0.87–0.89	< 0.01	0.79	0.79–0.80	< 0.01
Pará	1.00	—	—	1.00	—	—
Roraima	1.42	1.40–1.43	< 0.01	1.26	1.25–1.27	< 0.01
Rondônia	1.36	1.36–1.37	< 0.01	1.50	1.49–1.51	< 0.01
Year of case notification						
2004	1.00	—	—	1.00	—	—
2005	1.09	1.08–1.10	< 0.01	1.06	1.05–1.07	< 0.01
2006	1.19	1.18–1.20	< 0.01	1.13	1.12–1.14	< 0.01
2007	1.07	1.07–1.08	< 0.01	1.11	1.10–1.12	< 0.01
2008	1.04	1.03–1.05	< 0.01	1.10	1.09–1.11	< 0.01
2009	1.11	1.10–1.12	< 0.01	1.14	1.13–1.15	< 0.01
2010	1.11	1.10–1.12	< 0.01	1.12	1.11–1.13	< 0.01
2011	1.00	0.99–1.02	0.41	1.19	1.18–1.21	< 0.01
2012	1.07	1.06–1.08	< 0.01	1.44	1.42–1.47	< 0.01
2013	1.03	1.02–1.04	< 0.01	1.40	1.37–1.42	< 0.01
Socioeconomic variables						
Level of schooling						
No schooling–incomplete 5^th^ grade	1.31	1.30–1.32	< 0.01	1.20	1.19–1.22	< 0.01
Completed 5^th^ grade–9^th^ grade	1.06	1.05–1.08	< 0.01	0.96	0.95–0.97	< 0.01
Partial high-school to beyond	1.00	—	—	1.00	—	—
Not applicable	1.58	1.56–1.60	< 0.01	1.17	1.15–1.19	< 0.01
Not informed	1.67	1.64–1.69	< 0.01	1.42	1.40–1.44	< 0.01
Type of occupation						
Agriculture	1.11	1.10–1.12	< 0.01	1.06	1.05–1.07	< 0.01
Tourism	1.08	1.05–1.10	< 0.01	1.14	1.11–1.16	< 0.01
Livestock farming/crop production/hunting and fishing/bridge building/mining	1.00	—	—	1.00	—	—
Domestic	1.02	1.00–1.03	0.02	0.96	0.94–0.97	< 0.01
Prospector	0.94	0.93–0.96	< 0.01	1.03	1.02–1.05	< 0.01
Other	1.23	1.22–1.24	< 0.01	1.13	1.12–1.15	< 0.01
Not informed/not applicable	1.42	1.41–1.44	< 0.01	1.10	1.09–1.12	< 0.01
Malaria-related variables						
Type of malaria						
*Falciparum*	1.03	1.03–1.04	< 0.01	1.01	1.01–1.02	< 0.01
*Vivax*	1.00	—	—	1.00	—	—
Mixed	0.97	0.95–0.99	< 0.01	1.05	1.03–1.07	< 0.01
Other	0.51	0.45–0.58	< 0.01	0.67	0.59–0.76	< 0.01
Type detection						
Passive	1.00	—	—	1.00	—	—
Active	1.50	1.49–1.51	< 0.01	1.39	1.38–1.39	< 0.01

a Model adjusted for sex and parasite density, as well as for all the variables shown in the table.

***Source:*** Prepared by the authors from the study data.

## DISCUSSION

This is the first national study that identifies factors associated with the timely treatment of malaria in the BAR using a population-based analysis. Approximately 41.1% of cases began timely treatment (< 24 hours of symptoms onset). This result is potentially related to the continuous efforts to establish and maintain a broad network of malaria laboratories all over the BAR, even in the most remote areas. In 1999, there were just over 1 000 malaria laboratories in the area. In 2009, as a result of increased health care investment, the number of laboratories increased to more than 3 490, and the number health care professionals in malaria control and prevention reached 48 000 (15).

People receiving timely treatment were more likely to live in the states of Rondônia, Acre, and Roraima, to be less than 14 years of age, to be indigenous, to have a low level of schooling, and to be diagnosed via active detection. Approximately 65% of all cases reported during the complete time series (2004–2013) were notified in 2004–2008, while the last 2 years of study accounted for just 11% of all cases. Other studies have also pointed to recent reductions in malaria incidence in the BAR and the marked amplification of the areas with no malaria transmission (5, 16). International border areas where people live in vulnerable conditions and with poor access to health services (17–19) are exceptions.

Cases of *P. falciparum* showed the greatest reduction compared to *P. vivax*. Several factors may have contributed to its important decreasing trend, including climate changes, greater stabilization of urban conglomerations, increased distances between urban settings and the forest, changes and seasonal factors in the productive sector (e.g., mining and fish farming), and increased single crop production in the area (5, 20). In particular, the drop in the incidence of *P. falciparum* might be related to the introduction of the artemisnin-based combination therapy (21). Artemether-lumefantrine was shown to be an efficacious, safe, and convenient treatment for *P. falciparum* malaria in highly drug-resistant parts of South America (22). Collaborative efforts among municipalities, the states, and the Ministry of Health involving malaria prevention and control measures, including scaling up access to diagnosis and treatment, the distribution of insecticide-treated mosquito nets, and other vector control measures may also have been key to successful outcomes in malaria control (2, 5). In this regard, one of the important control measures adopted recently by the malaria program in Brazil is shortened time-totreatment (23).

Residents of the states of Acre (OR = 1.56), Rondônia (OR = 1.50), and Roraima (OR = 1.26) had a greater likelihood of timely treatment than those in Pará, while those in Amapá and Amazon had a lower likelihood of timely treatment. Nevertheless, this difference might be related to the complexity involving access to health care due to the expansive geographical areas of these states (730.6 km^2^ and 395.1 km^2^, respectively), compared to Acre (49.5 km^2^) and Roraima (40.6 km^2^) (24). Rondônia has achieved excellent results in combating the disease by means of malaria prevention and control policies based on rapid diagnosis and timely treatment, application of vector control measures (distribution of insecticide-treated mosquito nets), and rapid detection of epidemics (15, 25). Evaluation studies may be necessary to identify determinant factors associated with this positive outcome to help those with less successful programs.

With regard to demographic characteristics, young individuals (0–14 years) were associated with greater odds of timely treatment. A dose-response relationship can be seen for age, i.e., the younger the patient, the greater the odds of receiving timely treatment, and the older the patient, the lower the odds. Explanations for this finding may be associated with younger age groups having lower immunity owing to low lifetime exposure to malaria, and consequently, more severe symptoms, and thus seeking health services quickly. In addition, parents tend to take their children for care as soon as the first symptoms appear. On the other hand, the elderly may have a reduced immune response, asymptomatic or oligosymptomatic cases, and thus, difficulty in making differentiated clinical diagnoses for malaria, which may be a barrier to malaria elimination (26). These hypotheses need to be examined in greater depth in future studies.

Timely treatment was also associated with indigenous patients (OR = 1.41) and those with very low schooling (from no schooling to the 5^th^ grade; OR = 1.20). These variables indicate vulnerable groups who are highly dependent on the Brazilian public health care system (SUS). SUS health professionals tend to be more alert to the malaria diagnostic than providers in the private sector (1), and are generally more widely available where there is greater socioeconomic vulnerability and exposure to malaria.

As expected, in this study, patients identified in active detection appear to be more associated with timely treatment (OR = 1.39; 95%CI: 1.38 – 1.39) than those identified via passive detection. This is because health workers who visit households are advised to offer immediate treatment for malaria to all patients with positive slide or rapid test results, both for symptomatic and asymptomatic cases. Another study found that active detection of malaria cases in endemic areas contributed to the sustainable control of the disease (27).

It is important to discuss the challenges to malaria control in the BAR as a result of the *P. vivax* recurrence (due to hypnozoite persistence) and due to asymptomatic persons, especially as related to *P. vivax* malaria. Routine, free malaria treatment in Brazil includes drugs to eradicate the latent forms of the parasite (hypnozoites). Even so, some relapse cases may occur. Additionally, the magnitude and transmission impact of the asymptomatic malaria cases in Brazil are controversial and may vary from very low prevalence to as high as 49% in remote BAR communities living with continuous transmission (28, 29). In both scenarios—hypnozoite and asymptomatic carriers—early treatment as a single strategy will not be sufficient to control *P. vivax* malaria; effective, active identification and treatment of positive cases may be necessary. Other authors have discussed the challenges regarding asymptomatic cases as a barrier to eliminating malaria in endemic areas (30). This issue should be addressed along with strategies to improve time to treatment.

### Limitations

Despite the robust structure of the SIVEP-Malaria and its recognized good data quality, there are still some limitations that may have impacted this study. Firstly, despite the thousands of laboratories and health professionals across endemic areas (15), a small number of malaria cases may not have been included in the database due to underreporting or misdiagnosis, a common issue for studies using secondary data from national databases. Asymptomatic cases could also be a source of underreporting, but for this study, these were not considered part of the target population. Secondly, each case notified in the database was considered to be a new episode of malaria. Consequently, an individual with more than one positive test could produce over-reporting; however, considering the geographic barriers in the BAR to health care access, over-reporting would be uncommon. Finally, although the race/color variable appears as a factor associated with timely treatment, race/color only began to be consistently reported in 2011, and its quality and coverage was improved afterwards. Therefore, analysis regarding this variable must be considered with caution.

### Conclusions

Early diagnosis and timely treatment are extremely important in interrupting the malaria transmission cycle, in addition to being a secondary prevention measure that prevents malaria cases from progressing to serious forms of the disease and death (23). In this study, timely treatment (starting within 24 hours of symptoms onset) was identified in approximately 40% of all malaria cases notified in 2004 – 2013. Factors associated with timely treatment were: being of a young age or elderly, living in the states of Acre, Rondônia or Roraima, having 2012 and 2013 as the year of notification, low level of schooling, and being identified via active detection.

Stemming from the findings of this study, two recommendations are to raise awareness of the importance of timely treatment, especially among individuals of middle/working age, residents of Amapá, Amazon, and Pará, and across the private health care sector where those with more schooling tend to seek health services; and to improve and increase active surveillance of malaria cases.

Identifying factors associated to timely treatment can strengthen the strategies for malaria control program, especially considering the expected impact on gametocyte availability for malaria vectors. This matter is particularly important because malaria-related hospitalization and death are highly avoidable through effective primary health care actions. Timely treatment provides hope for malaria control and for achieving the target of interrupting transmission in the BAR.

### Acknowledgements.

The authors wish to thank the National Malaria Control Program at the Ministry of Health of Brazil for providing access to the SIVEP-Malaria database.

### Disclaimer.

Authors hold sole responsibility for the views expressed in the manuscript, which may not necessarily reflect the opinion or policy of the *RPSP/PAJPH* and/or PAHO.
